# Effects of Pro-Cholinergic Treatment in Patients Suffering from Spatial Neglect

**DOI:** 10.3389/fnhum.2013.00574

**Published:** 2013-09-12

**Authors:** N. Lucas, A. Saj, S. Schwartz, R. Ptak, C. Thomas, P. Conne, R. Leroy, S. Pavin, K. Diserens, Patrik Vuilleumier

**Affiliations:** ^1^Neuroscience Department, Laboratory for Behavioral Neurology and Imaging of Cognition, University of Geneva, Geneva, Switzerland; ^2^Division of Neurorehabilitation, Department of Clinical Neurosciences, University Hospital of Geneva, Geneva, Switzerland; ^3^Division of Neurorehabilitation and Geriatrics, University Hospital of Geneva, Geneva, Switzerland; ^4^Plein Soleil Fondation, Lausanne, Switzerland; ^5^Unit of Acute Neurorehabilitation, Department of Clinical Neurosciences, University Hospital and University of Lausanne, Lausanne, Switzerland; ^6^Center of Affective Sciences, University of Geneva, Geneva, Switzerland

**Keywords:** spatial neglect, fronto-parietal, attention, cholinergic network, nicotine

## Abstract

Spatial neglect is a neurological condition characterized by a breakdown of spatial cognition contralateral to hemispheric damage. Deficits in spatial attention toward the contralesional side are considered to be central to this syndrome. Brain lesions typically involve right fronto-parietal cortices mediating attentional functions and subcortical connections in underlying white matter. Convergent findings from neuroimaging and behavioral studies in both animals and humans suggest that the cholinergic system might also be critically implicated in selective attention by modulating cortical function via widespread projections from the basal forebrain. Here we asked whether deficits in spatial attention associated with neglect could partly result from a cholinergic deafferentation of cortical areas subserving attentional functions, and whether such disturbances could be alleviated by pro-cholinergic therapy. We examined the effect of a single-dose transdermal nicotine treatment on spatial neglect in 10 stroke patients in a double-blind placebo-controlled protocol, using a standardized battery of neglect tests. Nicotine-induced systematic improvement on cancellation tasks and facilitated orienting to single visual targets, but had no significant effect on other tests. These results support a global effect of nicotine on attention and arousal, but no effect on other spatial mechanisms impaired in neglect.

## Introduction

Neglect patients typically fail to explore the left side of space. These symptoms are most frequently encountered after right hemisphere stroke (for review, see (Vuilleumier and Saj, 2013), and result from large lesions in fronto-parietal areas with extensive involvement of deep white-matter fibers (Doricchi et al., 2008; Verdon et al., 2010). A breakdown of spatial attention has been consistently put forward to account for many deficits encountered in unilateral spatial neglect (Kinsbourne, 1970b; Bartolomeo and Chokron, 2002). These patients typically present with an initial orienting bias toward stimuli in ipsilesional space (Kinsbourne, 1970a; D’Erme et al., 1992), together with a deficit in disengaging attention from these stimuli to reorient toward the left side (Gainotti et al., 1991; Bourgeois et al., 2012, 2013). This deficit can be explained in terms of a biased competition for attentional selection and conscious perceptual processing, with an advantage for ipsilesional sensory inputs at the expense of contralesional information. Neuroimaging studies in healthy subjects have further corroborated the hypothesis of right hemisphere specialization for controlling and reorienting attention in space (Gitelman et al., 1999).

In parallel, various lines of evidence indicate that the cholinergic system is also implicated in spatial attention (Voytko et al., 1994; Selden et al., 1998; Sarter et al., 2001). Studies in both animals (Voytko et al., 1994) and healthy humans (Witte et al., 1997) show that nicotine (a powerful cholinergic agonist) may increase selective attention and resistance to distractors; whereas cholinergic blockade (e.g., by scopolamine) can severely interfere with attention and increase distraction (see e.g., Bentley et al., 2003; Sarter et al., 2005; Mansvelder et al., 2006; Heishman et al., 2010). Numerous findings in rodents and primates point to a critical role of cholinergic inputs to cortical areas, which are conveyed by the basal forebrain cholinergic nuclei through widespread projections and act to enhance selective attention. Destruction of basal forebrain cholinergic neurons lead to severe impairments in focused attention (Voytko et al., 1994) and increased distracter vulnerability, an effect that seems to depend on cholinergic inputs to prefrontal cortex (Newman and McGaughy, 2008). Likewise, cholinergic deficits impair cue detection (Parikh et al., 2007), presumably subsequent to cholinergic losses in medial prefrontal cortex.

In humans, cholinergic pathways project to several cortical areas through discrete white-matter bundles traveling in the depth of human frontal and parietal lobes (Selden et al., 1998). Because of their anatomical location, it is likely that these pathways are often interrupted by large stroke lesions in patients with spatial neglect (Vuilleumier and Saj, 2013). These pathways are thought to provide modulatory inputs to fronto-parietal and sensory areas, acting on cortical synapses to boost signal-to-noise and prolong neuronal responses (Sarter and Bruno, 2000). A loss of cholinergic inputs to the cortex might potentially contribute to impaired attention and insufficient activation of sensory areas in these patients, in keeping with the fact that lesions in the white-matter tend to lead to more severe and persistent neglect (Samuelsson et al., 1997; Bartolomeo et al., 2007; Verdon et al., 2010; Corbetta and Shulman, 2011; Saj et al., 2012).

Recent functional brain imaging in healthy subjects further demonstrate that cholinergic drugs can modulate activity in frontal and parietal areas during spatial attention and working memory tasks (Lawrence et al., 2002; Bentley et al., 2004; Thiel et al., 2005; Giessing et al., 2006). In spatial orienting tasks, nicotine may also facilitate shifts of attention after “invalid cueing” on the opposite side (Thiel et al., 2005; Thiel and Fink, 2008), an aspect of attention typically impaired in patients with parietal lesions (Posner et al., 1984).

Thus, several lines of research converge to implicate the cholinergic system in attentional processes disrupted in spatial neglect, but no study so far investigated the effect of cholinergic drugs on a range of standard clinical neglect tests. Selective attention and reorienting of attention in space both are most conspicuously disrupted in spatial neglect, but also repeatedly reported to be modulated by cholinergic transmission in posterior parietal cortices (Witte et al., 1997; Murphy and Klein, 1998; Thiel et al., 2005). Moreover, nicotinic stimulation may also enhance sustained attention via inputs to prefrontal cortex (Hahn et al., 2003), and deficits in sustained attention are also common in neglect patients (Chatterjee, 1995; Robertson et al., 1998; Chatterjee et al., 1999). Therefore, brain lesions extending into white-matter regions traversed by cholinergic pathways (Selden et al., 1998) might exacerbate neglect deficits by disrupting cholinergic modulation of different attentional components. However, the role of a cholinergic component in neglect has not yet been systematically explored. To our knowledge, only one recent study was conducted where an oral gum with nicotine was administered to a group of nine chronic neglect patients (Vossel et al., 2010), showing a global effect on attention reorienting in a Posner cueing task. Other pharmacological treatment attempts in neglect patients have used dopaminergic (Fleet et al., 1987; Gorgoraptis et al., 2012) or noradrenergic (Malhotra et al., 2006) drugs, but with variable success.

In the present study, we predicted that attentional deficits associated with spatial neglect might partly be alleviated by a substitution of cholinergic loss through a pro-cholinergic drug. We hypothesized that deficits in attention in neglect patients, typically resulting from voluminous brain lesions extending widely into subcortical white matter, may often be combined with (or exacerbated by) a disruption of cholinergic transmission to cortical regions, even when the latter are spared by the lesion but deafferented from cholinergic inputs. In a proof-of-concept study, we tested the effect of a single-dose (10 mg) of transdermal nicotine patch on various symptoms of neglect using a double-blind placebo-controlled design. Based on previous research in both animals and humans, we expected some improvement in both lateralized and non-lateralized aspects of attention. In addition, we also performed an exploratory analysis of anatomical lesions to verify whether any treatment benefit would depend on particular components of the cholinergic pathways.

## Materials and Methods

### Participants

The patient group consisted of 10 patients (8 women, 2 men) suffering from spatial neglect after a first-ever unilateral right-hemispheric stroke (except patient 1, who presented with right neglect after a left-hemisphere stroke). They were recruited from a consecutive series of stroke patients admitted to Geneva University Hospital and Plein Soleil Foundation (Lausanne). All patients gave their informed written consent to participate in this study according to the local ethics regulation of Geneva and Lausanne University Hospitals. Patients were all right-handed (except one), with mean age of 69.1 years (range: 51.2–79.2), and showed both clinical and radiological evidence of single focal lesion to the right hemisphere due to stroke, involving the middle cerebral artery (MCA) territory in all cases; while they had no other serious concomitant illness. Most patients had partial (five quadranopia) or full (three hemianopia) visual hemifield cuts as determined by clinical examination using confrontation (subsidiary analysis showed no systematic influence of hemifield defects on performance or treatment response). Patients were examined 6.45 months post-stroke on average (range: 1–15 months). They were included only if they had stable vigilance and sufficient cooperation to undergo a testing session of 45 min, and showed stable symptoms of neglect as assessed with a standard battery of tests (Rousseaux et al., 2001; Azouvi et al., 2003), including cancellation, line bisection, compound-word reading, and two computerized tests for lateralized target detection and cued target detection (Table 1). Patients were excluded if they were currently smoking ≥1 cigarette/day, and any past history of smoking was systematically quantified and registered (Table 1).

**Table 1:** **Demographic and clinical data of the neglect patients**.

Patient	Sex	Age	Months post-accident	Cerebral vascular accident	Arterial territory	Visual field loss	Handedness	Sensory extinction	Smoking history	Nicotine side effects	Lesion volume (voxel)	Initial neglect severity (high:>group median; L:<group median)
hd	m	72.63	3.77	i	MCA	Yes	r	None	Nihil	No	498682	High
sh	f	51.18	7.83	i,h	ACA	No	r	None	Nihil	No	598567	Low
ro	f	59.45	5.37	i	MCA, ACA	Yes	l	None	Previously 30–40 UPA; stopped 5 years ago	No	496357	High
pa	m	68.20	1.73	i	MCA	Yes	r	None	Previously 60 UPA; stopped since accident	No	504154	High
fu	f	78.37	14.00	h	MCA, ACA	Yes	r	None	Nihil	No	Not available	Low
co	f	53.18	13.93	h	MCA, PCA	Yes	r	Tactile	Nihil	No	18841	High
sc	f	79.21	15.10	i	MCA	No	r	Tactile	Nihil	No	225924	High
ki	f	78.95	0.80	h	MCA	Yes	r		Nihil	Mild diarrhea in morning	131425	Low
lu	f	75.41	0.93	i	MCA	Yes	r	Visual and auditory	Nihil	0	391841	Low
go	f	74.58	1.00	h	MCA	Yes	r	visual and auditory	Nihil	0	89240	Low

f, Female; m, male; i, ischemic; h, hemorrhagic; MCA, middle cerebral artery; PCA, posterior cerebral artery; ACA, anterior cerebral artery; r, right; l, left; UPA, smoking habit magnitude (unity/packet/year).

### Material and procedure

The effect of a medium dose transdermal nicotine patch on attention performance was studied in a double-blind placebo-controlled within-subject design, where each patient participated in a four day sequence. On day 1, baseline performance was measured on a standardized battery, comprising eight neglect tests, to establish initial neglect severity. On day 2, patients received either an active nicotine treatment patch (Nicorette^®^, 10 mg) or a placebo patch, the order being randomly assigned to successive patients. After 24 h of rest on day 3, allowing a complete washing-out of the active agent (when given), the second patch was given, complementary to the one applied on day 2 (i.e., day 2: placebo → day 4: nicotine; or day 2: nicotine → day 4: placebo). On days 1, 2, and 4, neglect was assessed using a similar battery of visuo-spatial attention tasks. For each subject, the testing took place at the same time of the day, reducing any contamination by circadian fluctuation in attention.

Each subject was treated once (on either day 2 or day 4) with the pro-cholinergic agent (Nicorette^®^, 10 mg), always administered by patch. Active and placebo patches were visually identical (provided by Pfizer, Inc.). The patch was applied in the morning between 7 and 8 a.m. and removed around 6–7 p.m. Neuropsychological effects were assessed 6–8 h after the patch was applied, given that peak absorption is reached 5–10 h after application (Swiss Medical Compendium). During each session, possible negative side effects were systematically monitored with a checklist, listing all symptoms declared by the producer on a three-level scale (0 = no effect, 1 = minor effect, 2 = major effect).

**Figure 1:**
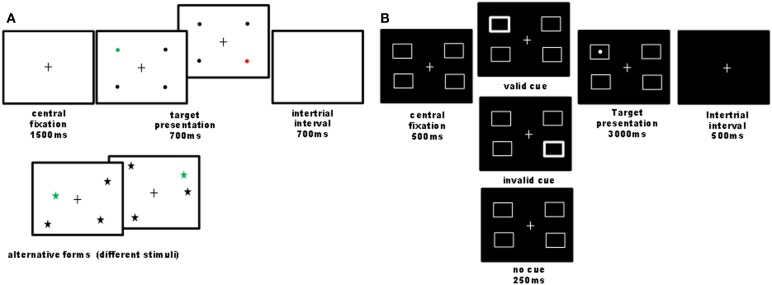
**Illustration of computer tasks**. **(A)** *Quadruplet detection task*: participants had to detect a single colored visual target among three black distracters and to report its color (e.g., red or green) as fast as possible by pressing one of two possible keys. On each trial, four stimuli were always presented, one in each quadrant, while the exact stimulus position within the quadrant was pseudo-randomly varied across trials. Different shapes and colors were used in different sessions (baseline, nicotine, placebo), counterbalanced across participants. Overall 44 trials were administered. In 90% of trials, a target was presented (half on the left and half on the right side); 10% of trials were catch trials, where no target was presented, in order to control for guess responses. This task was designed to assess visual detection in condition of stimulus competitions across the two hemifields, similar to extinction conditions (Vuilleumier and Rafal, 2000). The criterion for neglect presence on this task was a significant slowing of response latencies or increase in omission rates for targets on the left as compared to the right side. **(B)** *Cued detection task*: we designed a four-position variant of Posner’s paradigm with exogenous cues (24 trial by condition), where participants had to detect a lateralized target as quickly as possible, which could be preceded by a transient thickening of one of the four boxes or none. Validity and invalidity effects were calculated by comparing responses to targets following cues presented at the same or different locations. The cue validity was 50% to minimize the contribution of an endogenous allocation of attention. Patients reported detections by pressing on the computer space bar. The criterion for neglect presence on this task was a significant slowing of response latencies for targets on the left as compared to the right side.

The battery for assessing symptoms of spatial neglect was composed of eight different tasks probing visuo-spatial exploration, perception, and orienting (see Table 2 for details and Figure 1). For each of the tests, the stimulus support (paper-sheet or computer screen) was aligned with the midsagittal plane of the patient. The average assessment duration was around 45 min.

**Table 2:** **Tests used to assess neglect (Rousseaux et al., 2001) and dependent variables used for ANOVAs**.

Tests	Measure	ANOVA factor
**PAPER AND PENCIL TASKS**		
Bells’ cancellation task	Omission (left-right)	Target side
2 Versions	Search time	Contralesional vs. ipsilesional
Letter cancellation task	Omission (left-right)	Target side
3 Versions	Search time	Contralesional vs. ipsilesional
Shape cancellation task	Total omission (left-right)	
1 Version		
Compound-word reading task	Omissions/transformations (left-right)	Frame reference
2 Versions		Egocentric vs. allocentric
Line bisection (16 or 20 cm)	Deviation of the subjective midline<5%	% Of deviation
1 Version		
**COMPUTERIZED VISUAL TASKS**		
Lateralized visual detection task	Response latencies (left-right)	% Rates
Cued detection task (Posner’s paradigm)	Response latencies (left-right)	% Rates

Cancellation tasks: performance on the three cancellation tasks was evaluated using three different measures: number of omissions (per side of space), search duration (total time on the task, until the patient indicated to have finished the search or a maximum of 4 min), and the side of the first target canceled (right or left from the sheet midline).

Word reading task*:* the number of composite words omitted and the number of omissions/transformations of a composite-word part (typically its left part) were recorded for each side of space (right or left from the sheet midline).

Line bisection task: performance was measured as the deviation in mm of the subjective center relative to the true center of the line. Deviations exceeding 5% of total line length were considered pathological.

Quadruplet detection task: two dependent variables were measured, response latencies and detection rate (percentage of targets correctly reported for each side). To simplify our analysis and minimize multiple comparisons, both measures were collapsed into a single index of detection efficiency, by computing the ratio of the detection rate (number of hits) divided by the response latency (in milliseconds), multiplied by 100 to obtain speed-weighted percentage values. This quotient allows weighting the detection rate for a given condition as a function of the detection speed.

Cued target detection task (Posner’s paradigm): performance was evaluated in the same way as above, by computing a single efficiency score that combined the two dependent variables of detection rate (number of hits) and detection latencies (milliseconds), multiplied by 100 to obtain speed-weighted percentage values.

All tests (except star cancellation and line bisection) were given in different versions (different shapes or colors but with same spatial distribution and task structure) in different session (counterbalanced across participants), in order to minimize habituation or learning effects due to repeating the same tests.

### Data analysis

The performance scores from each task (Table 2) were submitted to a repeated-measure ANOVA with the within-subject factor TREATMENT CONDITION (3) (baseline, placebo, nicotine), plus more specific factors related to the task itself.

For the cancellation tasks: we ran mixed ANOVAs using the within-subject factors TARGET SIDE (2) (contralesional; ipsilesional), TREATMENT CONDITION (3) (baseline, placebo, nicotine), and the between-subject factor TEST (3) (letter cancellation, shape cancellation, Bells’ cancellation).

For the word reading task: repeated-measure ANOVAs using the within-subject factors TARGET SIDE (2) (contralesional; ipsilesional), and TREATMENT CONDITION (3) (baseline, placebo, nicotine), were conducted on the number of omissions/transformations per side of space relative to the midsagittal plane (egocentric frame of reference) and relative to the word-centered midline (allocentric frame of reference).

In the Quadruplet detection task and the Cued target detection task: to reduce variables in a concise but sensitive measure, we combined hit rates and reaction times to compute efficiency scores (i.e., hit/RT ratio), which were then entered into repeated-measure ANOVAs with the within-subject factors TARGET SIDE (2) (contralesional; ipsilesional), CUE TYPE (3) (invalid, no cue; valid), and TREATMENT CONDITION (3) (baseline, placebo, nicotine).

For the line bisection task: median deviations were calculated for each category of line length (16 and 20 cm) and for each patient, and then submitted to a repeated-measure ANOVA with the within-subject factor TREATMENT CONDITION (3) (baseline, placebo, nicotine).

Finally, we quantified initial neglect severity in all patients by calculating a global index of neglect deficits at baseline on day 1, dividing the number of tasks showing evidence of spatial neglect relative to the total number of tests given during this assessment, multiplied by 100. We distinguished patients with severe initial neglect [(USN+), above group median] vs. patients with moderate initial neglect [(USN−), below group median] by applying a median split on the group data. Changes during under placebo or nicotine were assessed relative to baseline performance.

### Lesion analysis

Brain lesions were confirmed by MRI or CT scans in seven and two patients respectively (for one patient only the neuro-radiological report was obtained) and reconstructed on axial slices using MRIcro (Rorden and Brett, 2000), following previously described methods (Verdon et al., 2010; Vocat and Vuilleumier, 2010; Saj et al., 2012; Vuilleumier et al., 2007). In two patients, we used CT scan to delineate the lesion site on a corresponding MRI template, as MRI could not be performed for clinical reasons. The lesioned areas were transformed to a 3D region-of-interest (ROI) corresponding to the lesion volume, and then normalized to a standard brain template using standard MRIcro and SPM methods (Ashburner and Friston, 1997; Ashburner et al., 1997). The normalized lesion ROIs were superimposed on a T1 MRI template and submitted to exploratory mapping analyses using MRIcro (Rorden and Brett, 2000), in order to examine the correlations between behavioral performance and anatomical extent of brain damage on a voxel-by-voxel basis. Firstly, we determined the average lesion overlap across all neglect patients. Secondly, we delineated critical lesion sites as a function of specific behavioral deficits in individual patients (e.g., neglect severity), or as a function of their sensitivity to nicotine treatment based on the observed improvement on neglect tasks.

## Results

### Good treatment tolerance

For the medium dose of nicotine administered here (10 mg), all patients in the present group showed a good treatment tolerance. Only two patients had a positive score for one item (diarrhea) on the negative symptom checklist. In one patient with a score of 2 on this scale (major symptom), the treatment was interrupted and the patient was not included into the study. The second patient presented a score of 1 (minor symptom) in the first few hours after patch application, but the symptom resolved after noon and the patient participated in the three sessions of the study without any further problem.

### Reduced neglect in cancellation tasks under nicotine treatment

**Figure 2:**
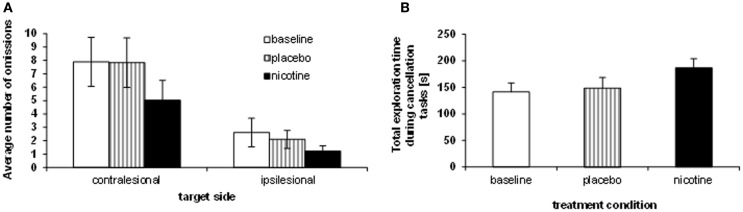
**Effects of treatment on neglect behavior**. **(A)** Sum of omissions averaged over the three cancellation tasks, separately for each target side (contralesional, ipsilesional). **(B)** Average total exploration time, across the three cancellation tasks (millisecond), showing longer search periods under nicotine as opposed to placebo and baseline performance.

We investigated visual exploration behavior on three different cancellation tasks (shape cancellation, letter cancellation, and Bells’ cancellation), which have different degrees of difficulty (as a function of the number of targets to be found, distracters to be ignored, and spatial crowding). First we compared the influence of treatment on target detection, as measured by the number of omissions in the three cancellation tasks, using a mixed 3 × 2 × 3 ANOVA, with the within-subject factors TREATMENT CONDITION (baseline, placebo, nicotine) and TARGET SIDE (contralesional, ipsilesional), plus the between-subject factor TEST (shape cancellation, letter cancellation, Bells’ cancellation). Performance significantly varied as a function of treatment, with the number of omissions being significantly reduced under the *nicotine* treatment (mean number of omissions: 2.93 ± 0.5) as compared to both *baseline* (4.95 ± 0.8) and *placebo* (5.14 ± 0.9) [main effect of TREATMENT CONDITION: *F*(2, 23) = 11.06, *p* < 0.0001]. As expected, the number of omissions on the left (contralesional) side (mean: 6.7) was globally higher than on the right [mean: 1.9; main effect of TARGET SIDE: *F*(1, 24) = 25.85, *p* < 0.0001]. This pattern of was similar for the three cancellation tasks [no main effect of TEST: *F*(2, 24) = 0.925, *p* > 0.05; no interaction with the other factors [TEST × SIDE: *F*(2, 24) = 0.75; *p* > 0.05; TEST × TREATMENT CONDITION: *F*(4, 48) = 0.4; *p* > 0.05]. The average number of *omissions* across the three cancellation tasks, calculated for each side separately and each patient, is plotted in Figure 2A.

While the reduction of omissions under nicotine was numerically greater on the left than the right side, the spatial asymmetry in omission distribution persisted in all sessions (no two-way interaction TREATMENT CONDITION × TARGET SIDE [*F*(2, 8) = 1.69; *p* > 0.05]. However, the reduction of omissions under nicotine was primarily driven by enhanced exploration toward the contralesional part of space, and omissions of ipsilateral targets were not entirely abolished. When investigating the effect of treatment condition on exploration for each side separately, a significant effect was found for *contralesional* targets only [main effect TREATMENT CONDITION: *F*(2, 8) = 9.92; *p* < 0.001], with fewer omissions under *nicotine* (mean number: 5.0 ± 1.5) as compared to both *baseline* [mean: 7.9 ± 1.8; *t*(9) = 4.67; *p* < 0.001] and placebo [mean number of omissions: 7.8 ± 1.9; *t*(9) = 3.92; *p* < 0.005]. The reduction of omissions on the ipsilesional side was not statistically significant [*F*(2, 8) = 1.39, *p* > 0.05].

Enhanced target detection during cancellation tasks went along with longer exploration times. Following standard clinical practice, patients were free to interrupt the task whenever they felt they had marked all targets, but given a maximum of 4 min. We computed the average exploration time across the different cancellation tests and submitted these data to a repeated-measure ANOVA with the within-subject factor TREATMENT CONDITION (baseline assessment, placebo, nicotine). Patients searched the cancellation arrays significantly longer under nicotine treatment (mean: 186.9 ± 51.6 s), as compared to baseline assessment [mean exploration time: 141.4 ± 50.6 s; *t*(8) = 3.5; *p* < 0.01] and placebo [mean: 148.7 ± 58.5 s; *t*(8) = 2.73; *p* < 0.05] [main effect TREATMENT CONDITION, *F*(2, 7) = 6.37; *p* < 0.01]. Figure 2B illustrates the average search times in each treatment condition, and shows these were significantly longer under nicotine treatment relative to both placebo and baseline. This increase was observed in all three cancellation tasks (Table 3).

**Table 3:** **Performance on individual cancellation tasks**.

	Bells cancellation			Letter cancellation		
	Baseline	Placebo	Treatment	Baseline	Placebo	Treatment
Mean	14	14.2	9	7.9	7.2	4.7
SD	7.7	10.2	5.4	8.4	4.5	5.3

**Figure 3:**
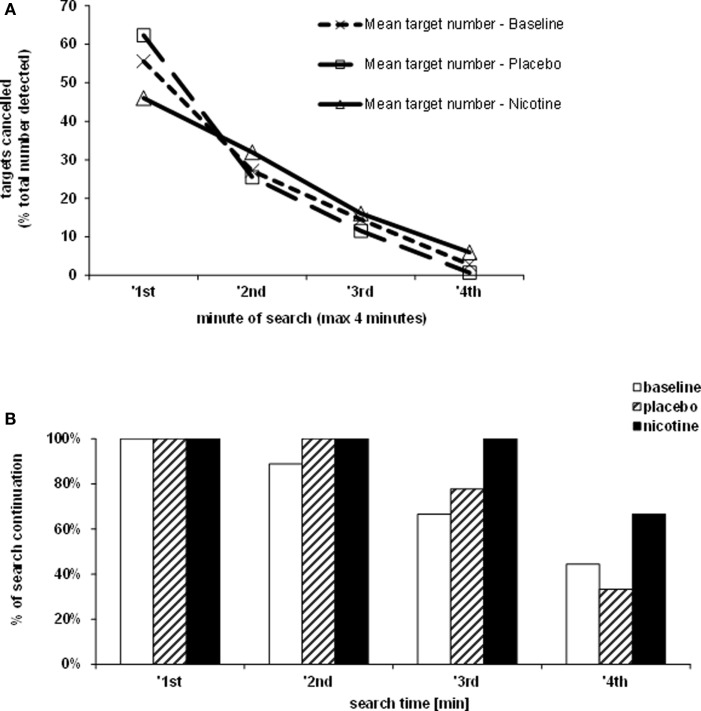
**Effects of treatment on exploration time**. **(A)** Target detection during the Bells’ cancellation task, plotted as the mean percentage of targets canceled per minute, relative to the total number of targets found by each participant in each test session. **(B)** Duration of search during the Bells’ cancellation task represented as the percentage of patients continuing to search for targets in time bins and each treatment condition. Search was self-terminated until a maximum allocated time of 4 min.

The rate of target detection over time was further examined in the Bells’ cancellation task since this task allowed tracking the number and location of detected targets across successive time-bins of 60 s (Rousseaux et al., 2001). Figure 3A shows that at baseline and under placebo, the majority of targets was found during the first minute, while only few additional items were detected in the subsequent time-bins. By contrast, under nicotine, the increase in detection rate was associated with a more regular detection rate over time. Thus, patients self-terminated search earlier in both the baseline and placebo conditions (i.e., no longer detecting any new target after 3 min in two third of cases), while they tended to continue search much longer when treated by nicotine (i.e., still exploring and detecting new targets until the time-limits of 4 min in more than half of cases; see Figure 3B).

On the other hand, the side of the first target canceled (in the three cancellation tasks) remained unchanged throughout the three treatment conditions.

### Enhanced performance in cued target detection

**Figure 4:**
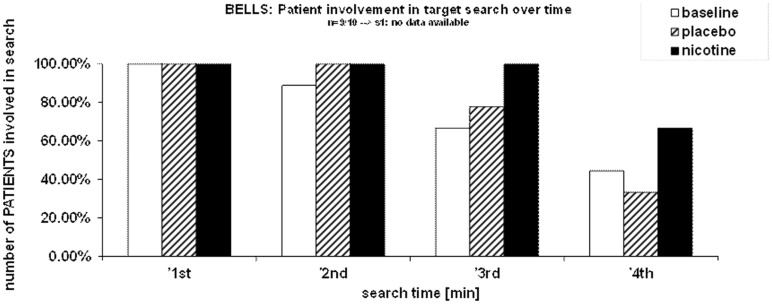
**Efficiency (hits/latencies) across different cueing conditions, for targets on both side of space relative to a mid-sagital plane separately**.

Effects of spatial cues on attentional orienting and subsequent target detection (Posner task) were analyzed in a 3 × 3 × 2 repeated-measure ANOVA with the within-subject factors TREATMENT CONDITION (baseline assessment, placebo, nicotine), CUE TYPE (invalid, no cue, valid), and TARGET SIDE (contralesional, ipsilesional). Attentional orienting significantly varied as a function of the cue type [main effect of CUE TYPE: *F*(2, 7) = 18.65; *p* = 0.0001], reflecting, as expected, a lower efficiency in the *invalid* condition (mean efficiency ratio of hits/RTs: 105.01 ± 9.56), relative to the two other cue conditions (all comparisons significant at *p* < 0.05; see Figure 4). Efficiency was intermediate in the *no-cue* condition (mean: 120.28 ± 12.12), and maximum in the *valid* cue condition (mean: 131.51 ± 12.12, significantly better than no cue, *p* < 0.005). Thus, the relative cost due to invalid cues and relative benefit due to valid cues both were reliably present in our patients. Note that the absence of an alerting signal in the no-cue condition was less harmful to performance than an invalid cue, consistent with the typical deficit in spatial attention associated with neglect.

As also expected, a robust difference in target detection efficiency was observed as a function of *target side* [main effect of TARGET SIDE: *F*(1, 8) = 31.86; *p* < 0.0001], with efficiency being overall better for targets in ipsilesional space (mean efficiency: 158.51 ± 10.83) as compared to targets in contralesional space (mean efficiency: 79.35 ± 15).

More importantly, attentional orienting was significantly influenced by the treatment [main effect TREATMENT CONDITION: *F*(2, 7) = 3.91; *p* < 0.05], with nicotine enhancing the efficiency for target detection (mean efficiency: 128.65 ± 11.54) relative to both the baseline assessment (mean efficiency: 108.12 ± 9.67; *p* < 0.05) and to the placebo condition (mean efficiency: 120.04 ± 13.93; *p* < 0.05). This improvement in efficiency was generally more important for the contralateral visual field (six patients detected the target faster under nicotine than placebo), in comparison with the ipsilateral field (only four faster under nicotine than placebo). No such improvement occurred under placebo as compared to baseline (*p* > 0.05).

Furthermore, nicotine treatment did not enhance detection in all cueing conditions similarly, as indicated by a significant two-way TREATMENT × CUE TYPE interaction [*F*(6, 3) = 2.05; *p* = 0.055]. Subsequently, to examine the critical planned comparisons, we computed 3 × 2 ANOVAs for the factors TREATMENT CONDITION and TARGET SIDE for each cue condition separately, which revealed that nicotine enhanced performance exclusively in the *valid* condition [main effect TREATMENT CONDITION: *F*(2, 7) = 4.42; *p* < 0.05] and in the *no-cue* condition [*F*(2, 7) = 3.46; *p* = 0.057], but not in the *invalid* condition [*F*(2, 7) = 1.87; *p* > 0.05]. However, these effects did not interact with TARGET SIDE. Thus, overall, detection efficiency was significantly enhanced by nicotine on both sides of space upon *valid* cues (mean: 146.76 ± 13.67) as compared to the baseline condition [mean: 116.84 ± 9.94; *F*(1, 8) = 16.22; *p* < 0.005], which in turn was similar to the placebo condition [mean: 130.94; ± 16.01; *F*(1, 8) = 1.32; *p* > 0.05]. A similar improvement of detection efficiency was found for targets presented without a preceding cue (*no-cue* condition, mean: 130.53 ± 11.53), relative to both the baseline (mean: 111.36 ± 11.54; *p* < 0.05) and the placebo condition (mean: 119.13 ± 15.07; *p* < 0.05), again irrespective of target side [interaction TREATMENT CONDITION × TARGET SIDE: *F*(2, 7) = 0.12; *p* > 0.05]. However, a formal test of the full three-way interaction (TREATMENT CONDITION × TARGET SIDE × CUE TYPE) did not reach significance [*F*(4, 6) = 0.543], which is likely to result from the small sample size relative to the number of conditions.

### No effect of nicotine on other tasks

No effect of nicotine treatment on neglect symptoms was found for the remaining tests. Nicotine did not induce any systematic amelioration on line bisection, a task where patients consistently showed rightward and highly variable deviation, irrespective of treatment condition (see Table 4).

**Table 4:** **Initial neglect severity in the baseline test session**.

	Sj nr	No tests done	No tests positive	% Test positive	BELLS omtot	% BELLS omtot	% Mean
HIGH initial neglect	7	8	8	100.00	28	80.00	90.00
	4	8	8	100.00	25	71.43	85.71
	1	8	7	87.50	11	31.43	59.46
	8	8	5	62.50	16	45.71	54.11
	5	3	3	100.00	19	54.29	77.14
LOW initial neglect	6	7	5	71.43	11	31.43	51.43
	11	6	4	66.67	6	17.14	41.90
	10	8	6	75.00	9	25.71	50.36
	9	8	4	50.00	8	22.86	36.43
	3	7	2	28.57	7	20.00	24.29

Neglect severity was determined by computing two different scores: (1) percentage of tests positive for neglect (based on asymmetries in response latency and/or accuracy in each test), relative to the total number of tests given to the patient; (2) percentage of omissions on Bells’ test, which is one of the most sensitive test for neglect and was given to all patients on all sessions. The same subgroups were constituted and the same results were obtained when defining the severity subgroup with either score.

No systematic effect was found for the composite-word reading task either. Nicotine did not induce systematic changes in the total number of words read on either side of the page. Neither did it modify the location of the first word read (egocentric neglect measures), nor did it reduce neglect dyslexia symptoms as determined by the number of omissions or transformations for the left part of compound words (allocentric neglect measures).

We note however that, in the present patient sample, *object-centered* neglect was consistently observed in one patient only (patient CF), for two different tests on different occasions (composite-word reading; shape cancellation, with discriminative target features on either their left or right side). No amelioration of these deficits was found under nicotine. Two other patients also showed signs of object-centered neglect but in the compound-word reading test only, and again none of them improved in this test under nicotine.

Finally, in the *Quadruplet detection* task, neither the number of misses nor the correct response time for contralateral targets were changed by nicotinic treatment. A 3 × 2 repeated-measure ANOVA was conducted on detection efficiency (ratio hits/RTs) with the factors TARGET SIDE (contralateral; ipsilateral) and TREATMENT CONDITION (baseline, placebo, nicotine), but only showed the neglect-specific spatial asymmetry [main effect of TARGET SIDE: *F*(1, 9) = 44.91; *p* < 0.0001]. Targets on the ipsilesional side were much more efficiently (more often and more rapidly) detected than targets on the contralesional side (mean efficiency: 151.4 ± 9.4 vs. 53.9 ± 13.2, respectively). However, nicotine did not reduce this asymmetry [main effect TREATMENT CONDITION: *F*(2, 8) = 1.88; *p* > 0.05; no interaction TREATMENT × TARGET SIDE: *F*(2, 8) = 0.32; *p* > 0.05].

### Nicotine treatment induces stronger improvement in patients with more severe neglect

In order to quantify the severity of neglect in our patient sample at the beginning of our study, we computed a score of baseline performance, based on the percentage of tests positive for neglect (relative to the total number of tests administered, since some patients did not complete *all* tests). As shown in Table 4, at baseline, before any treatment took place, patients with *severe initial neglect* omitted 45.7% of targets on the Bells cancellation task, whereas patients in the *moderate* initial neglect group omitted 25.7% of targets. Moreover, patients in the *severe* group showed positive neglect signs on 90% (range: 63–100%) of the tests (according to standard criteria for each test; see details in Materials and Methods section), whereas patients in the *moderate* group showed positive neglect signs on 58.3% (range: 28–75%) of the tests.

**Figure 5:**
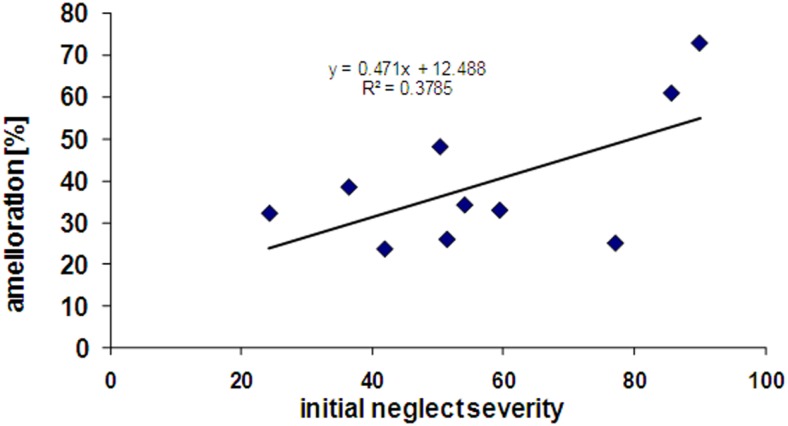
**Correlation between initial neglect severity (% tests failed at baseline) and extent of amelioration under nicotine treatment (% tests improved in the drug condition)**.

Interestingly, patients showing more severe initial neglect also showed better improvement under nicotine, as reflected by a positive correlation (*r* = 0.38) between the scores of initial neglect severity and the scores of amelioration by nicotine (see Figure 5). However, this correlation did not reach significance (*p* = 0.12, two-tailed) presumably due to the small sample size.

In addition, some patients were included at a relatively early stage post-stroke, whereas others were included at more chronic stages (range of days post-onset = 24–453). A moderate but again non-significant positive correlation between time since stroke onset (in number of days) and improvement was also found (*r* = 0.42, *p* = 0.10, two-tailed). This correlation nonetheless suggests that nicotine may exert some effects even at relatively late or chronic stages.

### Lesion analysis

Finally, we analyzed the patients’ lesions in order to examine any possible relationship between behavioral performance and the site or extent of brain damage. As our population sample was small, these analyses were essentially exploratory. Normalized lesion ROIs obtained from MRI reconstruction were used to determine the common overlap and differences between patients. In this sample, neglect severity did not correlate with lesion volume: the total number of voxels covered by lesion on the MRIcro brain template did not correlate with scores of initial neglect severity (*r* = −0.07).

**Figure 6:**
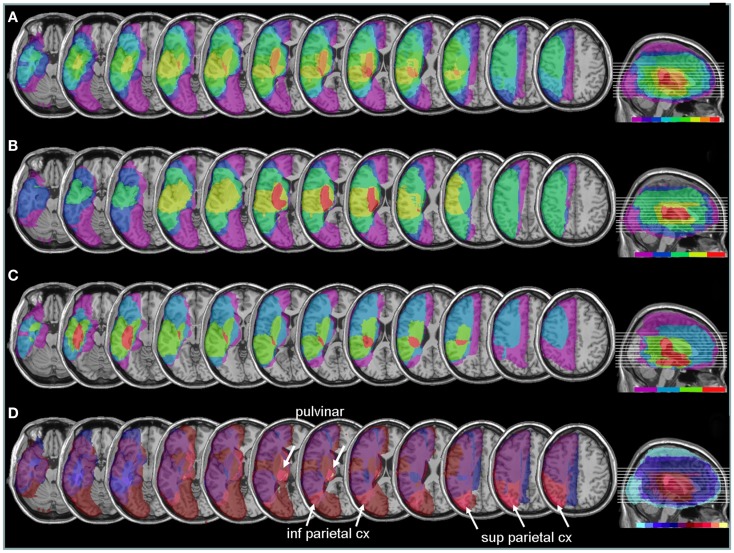
**Anatomical lesion analysis**. **(A)** Lesion overlap for the 9/10 patients for whom CT or MRI scans were available. Colors code for the number of patients with damage to a given area, ranging from purple for areas affected in one patient only, to red for areas affected in all patients. Brain regions most consistently damaged in our patients were located in the posterior limb of the internal capsule and deep parietal lobe (orange-red, corresponding to at least eight patients). **(B)** Lesion overlap in a subgroup of four patients with the most severe neglect deficits at baseline showing more extensive lesions in the right peri-sylvian and subcortical temporo-parietal junction. **(C)** Lesion overlap in the five patients with less severe neglect deficits at baseline, showing predominant damage in the temporal lobe and deep paraventricular white-matter. Colors code for the number of patients with damage to a given area (from 1 = violet to 5 = red). **(D)** Median split subtraction analysis, comparing the lesion in patients with severe vs. moderate neglect at baseline. Each color in the scale bar codes for a 16.67% frequency of lesion in one or the other group, except for the central purple color that represents −16.67 to +16.67%. More severe initial neglect correlated with more frequent damage to posterior parietal cortex and pulvinar (purple to yellow shades), while less severe neglect correlated with temporal white-matter damage (blue to turquoise shades).

Areas most commonly damaged in the present patient group were centered on the peri-sylvian subcortical white matter, extending posteriorly toward the inferior parietal lobe (Figure 6A). The maximal overlap involved the sub-insular white matter, including tracts of the external capsula and claustrum, in a position that is likely to disrupt the major afferents in the lateral cholinergic bundle projecting from the nucleus basalis of Meynert to the posterior frontal, parietal, and temporal cortices (Selden et al., 1998).

Comparing patients with more severe initial neglect to those with less severe neglect showed that the former had more extensive damage in the sub-insular white-matter regions and internal capsule, extending into dorsal caudate, putamen, and globus pallidus (Figure 6B); whereas less severe deficit was associated with lesions affecting the temporal lobe and the depth of the inferior parietal lobe, without basal ganglia involvement (Figure 6C). A direct contrast between these two subgroups using a voxel-wise subtraction analysis (Figure 6D) indicates that brain damage associated with *severe* initial neglect (purple–yellow) predominated in posterior parietal cortex and posterior thalamus (particularly in a region corresponding to the pulvinar). Whereas lesions associated with *mild* neglect were centered on the white matter of the inferior temporal lobe (dark blue–turquoise).

**Figure 7:**
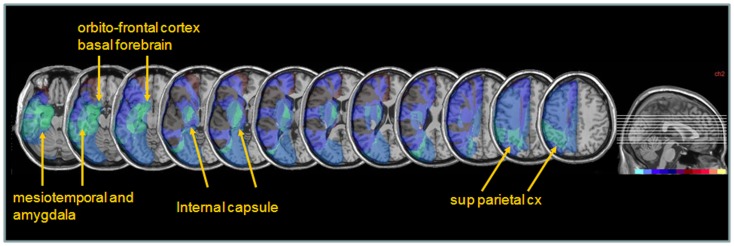
**Anatomical correlates of nicotine treatment efficacy**. Median split subtraction analysis, comparing the lesion in patients with the least important vs. the most important modulation of neglect (% tests failed across the whole battery or number of target omissions in Bells’ cancellation task) under nicotine relative to placebo. Each color in the scale bar codes for a 16.67% frequency of lesion in one or the other group, except for the central purple color that represents −16.67 to +16.67%. Lesions associated with the smaller improvement under nicotine were centered on subcortical white-matter fibers at the level of the basal forebrain, substantia innominata/sublenticular dorsal amygdala, as well as posterior parietal cortical areas.

Next, to determine whether different lesions accounted for different degrees of performance modulation by nicotine treatment, we distinguished patients showing a *low ameliorative effect* (*n* = 4) from those showing a *high ameliorative effect* (*n* = 5) under nicotine, based on a median split of improvement scores in each patient. Improvement was calculated as the difference in the global neglect severity score (% of positive tests) during nicotine treatment vs. baseline (cf. Materials and Methods section – Table 4). Note that the same patient subgroups were distinguished using a median split of changes in cancellation performance (difference in number of omission under nicotine vs. baseline). We then probed for the link between improvement and anatomical lesion sites using a voxel-wise subtraction analysis between patients with higher (*n* = 5) vs. lower amelioration (*n* = 4) under nicotine. As shown in Figure 7, reduced improvement was associated with lesions in the anterior mesial temporal lobe, with a maximum focus in dorsal amygdala (blue – turquoise voxels), as well as with lesions in the basal forebrain, internal capsule, and posterior parietal cortex (overlapping with intraparietal sulcus). Greater improvement was not found to correlate with consistent involvement of particular brain regions (dark purple and brown colored voxels).

## Discussion

The present study investigates the effects of pro-cholinergic treatment by nicotine in spatial neglect, using a series of classic neuropsychological tests and computerized measures of spatial attention. A significant improvement was found under nicotine for some tests but not others. This improvement tended to be more pronounced in patients with severe neglect, persisted in chronic stages, but depended on a relative sparing of parietal cortex, basal forebrain, and medial temporal lobe.

We employed a double-blind placebo-controlled within-subject design over three consecutive days, while spontaneous neglect recovery was unlikely to occur. Our major novel result is that a transdermal nicotine treatment with a single administration induced a consistent improvement of target detection and exploration behavior in three different cancellation tasks. Under nicotine, but not under placebo, the search performance of neglect patients was reliably improved, as reflected by a significant reduction of target omissions relative to both the placebo and baseline conditions. This improvement under nicotine was observed for targets on both sides of space, but with a more important reduction of omissions on the *contralesional* side. Nicotine also affected the duration of search behavior, by leading to more prolonged search times before terminating exploration and declaring all targets found (patients were free to continue or interrupt search until a maximum time limit of 4 min). This pattern suggests that nicotine enhanced the ability to progressively orient attention toward the contralesional side and/or disengage from previously explored locations on the ipsilesional side (Chatterjee et al., 1999), but without speeding target detection *per se*. Moreover, nicotine did not affect the *initial orienting bias* typically observed on cancellation tasks. Under nicotine, like at baseline or under placebo, patients invariably started their search on the ipsilesional (right) side of space (the first target canceled situated on the ipsilesional side of space).

By contrast, on tasks with a predominantly perceptual component, such as the line bisection and the quadruplet detection tasks, nicotine did not improve attentional biases of neglect patients. Both the extinction rate and detection latency asymmetries on the Quadruplet detection task remained unchanged, as did the rightward bias of the subjective midpoint during line bisection. A few previous studies have suggested a possible role for nicotine in boosting perceptual processing and representation in a *bottom-up* manner, either via enhanced selectiveness of thalamo-cortical transmission (Mooney et al., 2004; Disney et al., 2007) or through an amplification of early cortical visual processing (Stough et al., 1995; Thompson et al., 2000; Erskine et al., 2004), which would be expected to improve the detection of contralesional sensory stimuli in neglect patients (particularly in conditions of competition such as the quadruplet detection task here). However, such an effect of nicotine is not supported by the present findings, since detection efficiency in this task remained unchanged in our patients under the active drug treatment. Likewise, the distortion or compression in space representation underlying line bisection deficits (Bisiach et al., 1998) does not appear to be modulated by cholinergic function.

Finally, reorienting of spatial attention to the contralesional side subsequent to an invalid ipsilateral cue (i.e., Posner task), which is typically deficient in neglect patients (Bartolomeo and Chokron, 2002; Corbetta and Shulman, 2002), was not affected by nicotine in our study. However, we found an improvement in detection efficiency for targets presented after a valid cue or without a cue. Previous results from similar tasks in healthy human volunteers have been mitigated, with some studies reporting enhanced reorienting performance under nicotine with both endogenous (Thiel et al., 2005; Meinke et al., 2006) and exogenous cues (Witte et al., 1997; Murphy and Klein, 1998), while others failed to find reliable effects – with either exogenous (Meinke et al., 2006) or endogenous cues (Griesar et al., 2002; Meinke et al., 2006). A Posner task was also used to examine the effect of nicotine treatment in patients with spatial neglect in a recent study (Vossel et al., 2010), published after we reported our preliminary results elsewhere (Lucas et al., 2006). Results from this study showed that nicotine produced a non-specific speeding of RTs, without modulating the validity or invalidity effects of spatial cues, suggesting an influenced on tonic attentional processes like vigilance or sustained attention. These data accord with our own results, since we found that neither the detection rate nor the latency for reorienting to the contralesional side after invalid cues were improved.

Nevertheless, our results suggest an improvement in detection efficiency that was selectively observed for the uncued and validly cued targets. This improvement was spatially unspecific, i.e., not significantly lateralized to the contralesional or ipsilesional side. This improvement might reflect a nicotine-induced increase in cortical arousal and facilitation in processing task-relevant information, as reported by several behavioral studies after increased cholinergic levels through smoking or nicotinic drug (Knott et al., 1999; Gilbert et al., 2000). One study (Griesar et al., 2002) testing the effect of nicotine on alertness and covert orienting with endogenous cues reported similar findings in healthy non-smokers: participants showed a general improvement of latencies, in the absence of any spatially specific effect on orienting or reorienting of attention. Simultaneous EEG recordings also corroborated the hypothesis that the enhanced target detection was related to enhanced alertness. We note that, in our study, the absence of a similar improvement in the quadruplet detection task might possibly be due to the fact that that this task required a speeded discrimination, whereas the cued target detection task (Posner paradigm) required a simple detection response, and no-cue trials were unilateral without any competing distractors.

Consistent with our findings that nicotine may speed target information processing, a number of studies in different species have reported beneficial effects of nicotinic treatment on sustained attention (Trimmel and Wittberger, 2004; Spinelli et al., 2006). Therefore, we believe that the selective improvements in cancellation and cued target detection tasks in our patients might rely at least partly on an increase of sustained attention, possibly by enhancing arousal (Robertson et al., 1998) or general motivation factors (Mesulam, 1999), which are often impaired in neglect patients (Finke et al., 2012). In keeping with this assumption, both tasks for which neglect patients showed improvement were also the two tests with the longest duration: cued target detection task (7.5 min) and cancellation tasks (4 min); unlike the remaining tasks which all took on average ≤2.5 min.

It is important to note that, under nicotine, the improved exploration of contralesional space during cancellation tasks went along with longer search times. Patients were instructed to “*search and cancel targets, until they felt that there were no more targets left unmarked*.” This suggests that, across the three cancellation tasks, nicotine apparently influenced the patient’s criterion to stop search. This could also be related to sustained attention or motivational factors, in accord with putative cholinergic functions.

### Neural substrates for nicotinic effects on attention

Neurobiology research suggests that cholinergic neurons in the basal forebrain are critically implicated in the analysis and/or response to the behavioral significance of sensory cues (Wilson and Rolls, 1990). In particular, the basal forebrain cholinergic corticopetal system has been hypothesized to operate as a relay for modulatory influences from the amygdala and other limbic areas (such as the dopaminergic reward pathways, see Rice and Cragg, 2004), which are exerted on cortical sensory areas (Bentley et al., 2003) as well as on other cortical systems involved in attention and top-down executive control (Sarter et al., 2005). Increased nicotine tone may thus enhance signals of behavioral saliency to amplify activity in visual cortices and/or boost fronto-parietal regions generating spatial or attentional saliency maps.

Indeed, neuroimaging studies after nicotine administration have shown consistent modulations of parietal and frontal activity. Using a working memory task in ex-smokers, Ernst et al. (2001) found that improved performance under nicotine depends on prefrontal and parietal cortices bilaterally. In non-smoking subjects, Kumari et al. (2003) also showed higher activation of parietal and frontal areas during a working memory task. Regarding attentional processes, several studies reported modulations of fronto-parietal cortex but with either reduced (Thiel et al., 2005; Vossel et al., 2008) or increased activation in attention-related networks (Lawrence et al., 2002). Using a sustained attention task, Lawrence et al. (2002) found that activity changes in bilateral inferior parietal cortices, precuneus, thalamus, and caudate nucleus mediated the behavioral costs of smoking abstinence and benefits of nicotine replacement on the sustained attention performance.

In sum, our data converge with these studies to suggest that nicotine might improve neglect by boosting the representation of behaviorally relevant target stimuli (as opposed to distracter stimuli), and by promoting sustained attention over longer periods of time, with such effects arising independently from spatial biases due to unilateral damage in the frontal and/or parietal attentional network.

### Distinct motivational and attentional effects of nicotine

An effect of nicotinic stimulation on arousal or motivational systems, rather than on spatial attention systems, is supported by two main findings: firstly, despite the fact that nicotine reduced omissions in cancellation tasks more markedly for the contralesional side, and non-significantly for the ipsilesional side, a formal statistical test for this difference remained non-significant (no reliable two-way interaction TARGET SIDE × TREATMENT CONDITION). Moreover, a differential improvement per side may partly depend on the number omissions committed at baseline (since few omissions at the beginning would result in a low potential for improvement; but numerous omissions would provide a high potential for improvement). In the same line, nicotine effects on the *Cued target detection* task arose for the *valid-cue* and the *no-cue* condition in both the contralesional and ipsilesional sides. As discussed above, these behavioral effects suggest a global facilitation without any spatially specific component. Such global effects might accord with other findings that neglect can be improved by transient arousal (Finke et al., 2009) and motivational incentives conveyed by reward (Malhotra et al., 2013; Mesulam, 1985) or reward learning (Lucas et al., 2013).

Secondly, the results of our exploratory anatomical analysis indicated that the nicotine-induced change in neglect behavior appeared to be lower in patients whose lesion extended into the basal forebrain region just dorsal to the amygdala and into the internal capsule, as well as (to a lesser degree) into more posterior parietal regions (see Figure 6). Though these interpretations must be taken with caution because of the small sample size and inherent variability of lesions in stroke patients, our data suggest that an effective impact of nicotine treatment might critically dependent on the integrity of the cholinergic projection systems in the basal forebrain region (Selden et al., 1998). Hence, patients suffering from lesions encompassing on this structure or its projections to parietal areas would show little amelioration under nicotine (unlike patients in whom these areas are spared). Although cholinergic enhancement due to nicotine might also take place at the synaptic levels in the target cortical zones, a preservation of some projections pathways from basal forebrain might be important to provide task-related modulations and more effective cholinergic activity in attention-demanding situations.

In addition, however, damage to superior parietal cortex was also found to reduce the benefit of nicotine (see Figure 7). This negative correlation accord with the notion that the pharmacological effect of nicotine on spatial attention might be mediated by modulation of parietal areas in healthy people (Thiel et al., 2005), and the related finding of Vossel et al. (2010) that such benefits might be absent in neglect patients when their lesions extent to parietal lobe. In our study, a sparing of superior parietal cortex in patients showing greater improvement in cancellation performance under nicotine suggests that this effect might depend on a boosting of attentional mechanisms subserved by these parietal regions (Corbetta and Shulman, 2011), which control endogenous orienting and promote active exploration.

Finally, we found that patients with more severe neglect at baseline tended to show greater amelioration effects under nicotine. Comparisons between initial neglect severity and changes under nicotine revealed a remarkable correlation between severity and nicotine benefit (*r* = 0.58). This relation may reflect the fact that more severe deficits gave greater opportunity to observe changes, or that more severe neglect symptoms may be associated with greater damage to brain systems mediating arousal functions sensitive to nicotine stimulation (Finke et al., 2012). We also note however that, in the present study, severe neglect was associated with more frequent damage to parietal areas, in line with previous anatomical findings in Mort et al. (2003) and Saj et al. (2012), as well as subcortical areas such as the pulvinar (Karnath et al., 2002). Future studies with larger patient groups are necessary to determine whether only patients with subcortical forms of neglect may benefit from pro-cholinergic therapy, and which aspects of neglect behavior may be improved in different patients as a function of their lesion sites.

## Conclusion

To sum up, our study investigated the effects of pro-cholinergic treatment by nicotinic receptor stimulation in spatial neglect. Our results converge with those of a parallel study using nicotinic gums (Vossel et al., 2010) but also extend them by better delineating the range of improvement or non-improvement in different tasks. Another recent pharmacological study using the norepinephrine-enhancer guanfacine observed very similar results in two neglect patients, but not a third (Malhotra et al., 2006). In this study, the norepinephrine drug also improved search in multi-target displays, with better detection going along with prolonged search times, in the absence of any improvement for speeded tasks tapping into more perceptual functions. It is intriguing that globally similar effects were obtained on a similar cancellation tasks using different kinds of drug, targeting the norepinephrine in the latter study, and the cholinergic system in ours. Moreover, the effect was quantitatively similar to Malhotra et al. (2006) with a ∼20% of change in target detection. Although originating from different structures in brainstem (locus coeruleus for NE) and basal forebrain (Meynert nucleus cholinergic for ACH), cortical projections of these two neuromodulatory systems have partly overlapping distribution predominating in prefrontal and parietal areas (Russell et al., 2013). However, these two systems might modulate cortical arousal and information processing in different ways. ACH release in the cortex is increased both prior and during sustained attention demands, with further increase in response to distracters, presumably serving to enhance signal to noise of behaviorally relevant targets (Himmelheber et al., 2000; Klinkenberg et al., 2010). Conversely, tonic levels of NE are lower during search, allowing greater selectivity, but with phasic peaks to target detection, while higher tonic levels are present under state of inattentiveness in order to facilitate response to new or unexpected information (Aston-Jones and Cohen, 2005). Further studies would be useful to directly compare both drugs in the same patients and across various tasks. Variations in lesion site or extent might also lead to different therapeutic responses in different patients. Here, we found that subcortical limbic structures may be critically involved in the mediation of improved orienting and target detection during exploration, as nicotinic effects were reduced in patients whose lesions extended in mesial temporal lobe and basal forebrain, as well as internal capsule and posterior parietal cortex (see Figure 7). It remains to be seen if these patients showing little effects under nicotine would show greater benefits from guanfacine, and vice versa.

Future studies should also explore the possible benefits from more prolonged treatment with pro-cholinergic agents, compare them with other drugs such as noradrenergic or dopaminergic agonists, as well as use a combined stimulation of both the nicotinic and muscarinic cholinergic receptors. For example, in the treatment of Alzheimer’s disease, other pharmacological cholinergic agents such as donepezil (an acetylcholine esterase inhibitor) are already used with a certain success, possibly leading to positive behavioral effects via improvement of attentional functions (Levy et al., 2000; Mansvelder et al., 2006; Heishman et al., 2010). These benefits of pro-cholinergic drugs in dementia and other clinical conditions (e.g., head injury) further show that such treatment may improve attentional deficits even in the absence of spatial neglect, perhaps by acting upstream on global arousal and motivational processes. It remains to be determined whether beneficial attention effects might also be obtained in neglect patients with such treatment, particularly when they present with low arousal or deficits in sustained attention.

## Conflict of Interest Statement

The authors declare that the research was conducted in the absence of any commercial or financial relationships that could be construed as a potential conflict of interest.
