# Erratum: Effects of pro-cholinergic treatment in patients suffering from spatial neglect

**DOI:** 10.3389/fnhum.2013.00700

**Published:** 2013-10-22

**Authors:** Frontiers in Neuroscience Editorial Office

**Keywords:** spatial neglect, fronto-parietal, attention, cholinergic network, nicotine

Dr. Schnider should have been removed from the authors list. The correct authors list should be as follows: Lucas N, Saj A, Schwartz S, Ptak R, Thomas C, Conne P, Leroy R, Pavin S, Diserens K and Vuilleumier P.

